# Large Gastric Perforation in Carmi Syndrome: A Morbid Complication in a Rare Association

**Published:** 2012-10-01

**Authors:** M Joshi, L Krishnan, S Kuruvila

**Affiliations:** Department of Pediatric Surgery, Pondicherry Institute of Medical Sciences, (PIMS), Kalapet Pondicherry, India.; 1 Department of Pediatrics, Pondicherry Institute of Medical Sciences, (PIMS), Kalapet Pondicherry, India.; 2 Department of Dermatology, Pondicherry Institute of Medical Sciences, (PIMS), Kalapet Pondicherry, India.

**Keywords:** Epidermolysis bullosa, Congenital pyloric atresia, Carmi syndrome, Gastric perforation

## Abstract

The association between epidermolysis bullosa (EB) and congenital pyloric atresia (CPA) named Carmi Syndrome is rare. We report unusual and morbid complication of gastric perforation resulting in peritonitis in a preterm neonate born with Carmi Syndrome.

## INTRODUCTION

Congenital Pyloric Atresia (CPA) and Epidermolysis Bullosa (EB) has reported incidence of one in 100,000 live births and one in 300,000 live births respectively [1, 2]. This rare association having autosomal recessive inheritance was first described by Swinbume and Kohler in 1968 and is now known as Carmi syndrome [1, 3]. Exact incidence of this rare association is not known. We report a preterm neonate born with Carmi Syndrome who had gastric perforation resulting in peritonitis. Isolated case reports of gastric perforation in CPA are available in literature [4-6]; extensive literature search could reveal only one neonate with Carmi Syndrome hitherto who had gastric perforation [7].

## CASE REPORT

An out born male preterm (32 week's gestation) weighing 1.2 kg at birth was seen on day three of life with severe abdominal distention. On examination, the baby was lethargic and had distended and tense abdomen. Abdominal skin was erythematous. Bowel sounds were absent. X-ray abdomen on day two showed prominent gastric bubble with no distal gas. Repeat X-ray showed pneumoperitoneum and gas-filled distended stomach with distal air pockets (Fig. 1).

**Figure:**
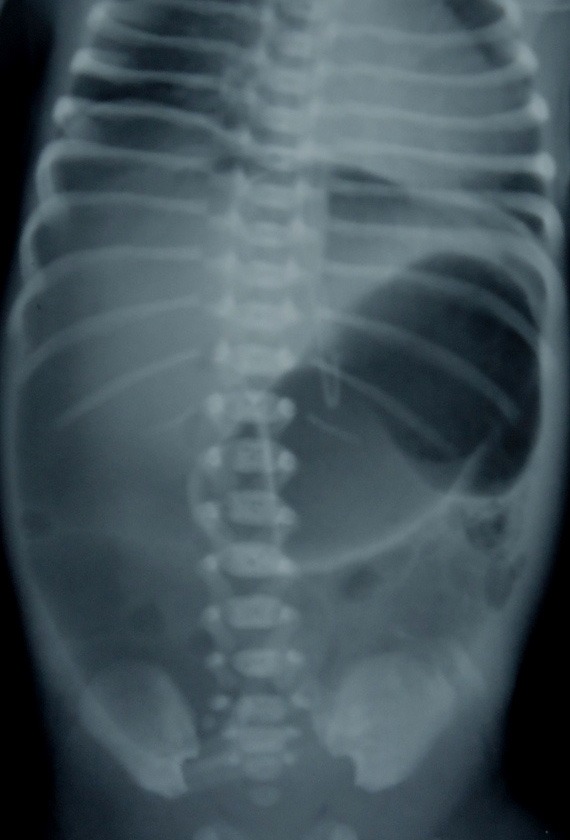
Figure 1: Plain X-ray abdomen showing pneumoperitoneum with loculated air pockets and distended stomach.

Emergency laparotomy was performed after initial stabilization of vital parameters; it revealed a large full thickness gastric perforation along greater curvature with unhealthy margins. Peritoneal cavity had loculated collections sero-mucoid fluid, and yellowish mucous flakes. Stomach was inspected from inside. It revealed type one pyloric atresia. Pyloric diaphragm was excised and wide Heineke-Mikulikz pyloroplasty was performed. Peritoneal lavage and repair of gastric perforation was done; the stomach was additionally decompressed with gastrostomy. The baby was electively ventilated. Initial 24hrs were uneventful. On day two, the baby developed bullous lesions over trunk and extremities, with peeling off of skin (Fig. 2).

**Figure:**
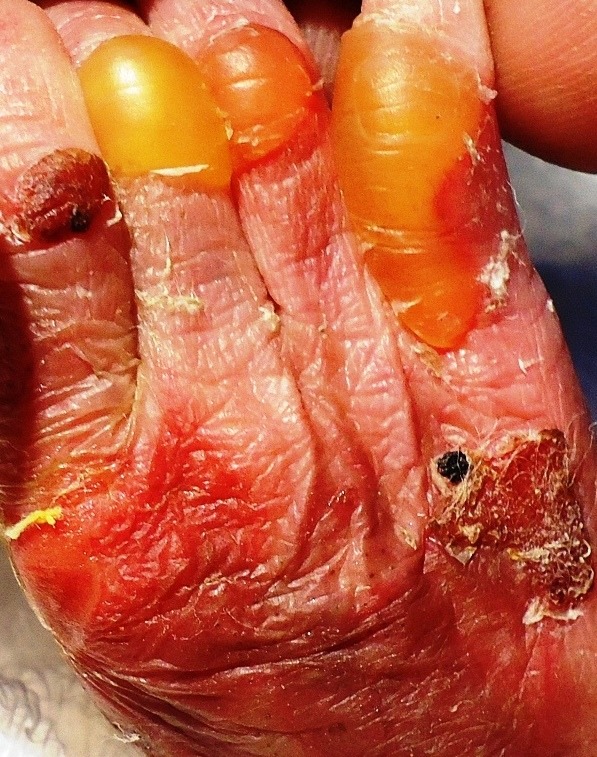
Figure 2: Bullous lesions with skin peel-ing over extremity of baby.

Epidermolysis Bullosa (EB) was suspected and skin biopsy was taken. Specimen was sent for indirect immuno-fluorescence with antigen mapping at a different centre. Report confirmed the diagnosis of junctional EB. Peritoneal fluid culture grew Klebsiella. On day five of life, the baby developed leakage. Re-exploration revealed leakage from the previous gastric repair with ischemic margins and narrowing of pyloroplasty lumen. Gastroduodenostomy was performed and perforation was repaired after excising unhealthy margins. Oral feeding was delayed due to prolonged paralytic ileus and sepsis. Total leukocyte counts were 19 x 103/ dl; CRP was positive. The parents took the baby away against medical advice on postoperative day eight. At the time of discharge, few old skin lesions over face showed evidence of healing, but new blebs were still appearing over trunk.

## DISCUSSION

Although perforation of the gut proximal to an obstruction is possible and is often seen, such a complication has not been reported even in the large series of Carmi Syndrome [1, 8]. Rarely, perforation of pharyngo-esophageal junction, serosal tear of the stomach and full-thickness gastric perforation have been reported in CPA patients [3, 7, 9].

Though exact etiology is not known, accepted theory is that Carmi Syndrome is genetically determined and their occurrence together is due to pleiotropic expression of a single gene or action of two closely related genes [1]. Intrauterine sloughing of mucous membrane with subsequent healing by fibrosis in EB is also suggested [1]. This is supported by reports of multiple associated atresias and acquired pyloric atresia [1]. 

Trauma by vigorous nasogastric tube insertion or aggressive bag and mask ventilation to manage apnea has been implicated as a cause of gastric perforation [9]. In our case, day two X-ray was suggestive of pyloric obstruction. We believe that increased intra-gastric pressure due to distal obstruction and continued oral feeding could have been the precipitating factor for perforation in this case. Positive pressure ventilation could not be documented. Surgical treatment in isolated CPA is corrective. Modalities available are Heineke-Mikulikz pyloroplasty for type one membranous atresia and gastro-duodenostomy for type two and three variety [1]. Isolated pyloric atresia has good prognosis after successful surgery [1]. However, results in babies with EB and complications like gastric perforation are not encouraging. Postnatal delayed presentation may cause complications of perforation. Peritonitis due to perforation leads to high morbidity. Sepsis and fluid loss are major cause of mortality in EB [1, 8]. Nutritional deprivation due to prolonged paralytic ileus in postoperative period worsens the situation. Parenteral nutrition also holds high risk due to skin peeling off near port of entry and colonization leading to sepsis. Overall mortality in isolated CPA is more than 50% [1]. No exact mortality data exist for Carmi Syndrome. 

Perforation peritonitis in CPA is preventable complication. Newborn with intolerance to feeds and non-bilious vomiting should be investigated early. Presence of single bubble with no distal gas shadows in X-ray should alert the physician. Further bag and mask ventilation and feeding should be withheld. Prenatal ultrasound in high risk cases with previous infantile death is valuable for early diagnosis. CPA may be diagnosed at tenth week by high resolution ultrasound [1]. Polyhydramnios, dilated stomach and esophagus are suggestive features [9]. Analysis of DNA extracted from fetal cells by chorionic villus sampling at ten to 12 weeks gestation is a safe and reliable alternative test. When available, fetal skin biopsies by fetoscopy is also diagnostic of EB at 19 weeks [1]. 

We emphasize need for detailed prenatal counseling to parents with previous history of new-born death and skin lesions. Although rare, but in view of high incidence of mortality, cases with clinical and radiological suspicion of Carmi Syndrome should be referred timely to surgical unit to prevent perforation related complications. This may possibly change the outcome of illness.

## Footnotes

**Source of Support:** Nil

**Conflict of Interest:** None
